# Linear Decay of Retrotransposon Antisense Bias across Genes Is Contingent upon Tissue Specificity

**DOI:** 10.1371/journal.pone.0079402

**Published:** 2013-11-14

**Authors:** Sara Linker, Dale Hedges

**Affiliations:** 1 Hussman Institute for Human Genomics, Dr John T. Macdonald Foundation Department of Human Genetics, Miller School of Medicine, University of Miami, Miami, Florida, United States of America; 2 Division of Human Genetics, Department of Internal Medicine, The Ohio State University, Columbus, Ohio, United States of America; Ben-Gurion University, Israel

## Abstract

Retrotransposons comprise approximately half of the human genome and contribute to chromatin structure, regulatory motifs, and protein-coding sequences. Since retrotransposon insertions can disrupt functional genetic elements as well as introduce new sequence motifs to a region, they have the potential to affect the function of genes that harbour insertions as well as those nearby. Partly as a result of these effects, the distribution of retrotransposons across the genome is non-uniform and there are observed imbalances in the orientation of insertions with respect to the transcriptional direction of the containing gene. Although some of the factors underlying the observed distributions are understood, much of the variability remains unexplained. Detailed characterization of retrotransposon density in genes could help inform predictions of the functional consequence of *de novo* as well as polymorphic insertions. In order to characterize the relationship between genes and inserted elements, we have examined the distribution of retrotransposons and their internal motifs within tissue-specific and housekeeping genes. We have identified that the previously established retrotransposon antisense bias decays at a linear rate across genes, resulting in an equal density of sense and antisense retrotransposons near the 3′-UTR. In addition, the decay of antisense bias across genes is less pronounced among tissue-specific genes. Our results provide support for the scenario in which this linear decay in antisense bias is established by natural selection shortly after retrotransposon integration, and that total antisense bias observed is above and beyond any bias introduced by the integration process itself. Finally, we provide an example of a retrotransposon acting as an eQTL on a coincident gene, highlighting one of several possible avenues through which insertions may modulate gene function.

## Introduction

Retrotransposons (RT) make up approximately half of the human genome [1], with Alu and L1 elements comprising the two most active elements in modern humans. Although host mechanisms inhibit their amplification rate [2]–[4], human RTs maintain an insertion rate of approximately 1 in 20 and 1 in 270 human births for Alu and L1 respectively [5], [6]. While many of these insertions have no apparent pathological consequence, a subset are known to have deleterious effects [7], [8]. It has been proposed that these effects, in part, drive the observed non-uniform distribution of RTs across the genome [9]–[11]. This non-random distribution has been documented for both the Alu as well as the L1 retrotransposon. Alu and L1 initially insert randomly across the genome, but they tend to accumulate in GC-rich and GC-poor regions, respectively [12], [13]. Understanding the factors associated with this observed distribution could help to predict how the functional impact of a given RT insert is determined by its surrounding genomic context.

RTs contain DNA motifs that can act as binding sites for polymerases [14]–[16], polyadenylation enzymes [17], transcription factors [18], and other potential regulatory proteins. In extreme cases, an insertion into the coding region of a gene could result in the complete disruption of the gene [7]. However, when retrotransposons integrate, they may also impact transcriptional regulation [17], [19], [20] and/or chromatin structure [21], [22]. For example, Wang et al. showed that different fragments of the L1-ORF2 modulated transcription termination of upstream GFP expression [23], demonstrating functional impact of the L1 sequence on gene transcription without a direct disruption of coding sequence. The functional consequences of RT retrotransposition are not always deleterious, however, and in some instances they have led to the evolution of mammalian-specific [24] and human-specific traits [25]. On a genome-wide level, retrotransposition has been the source of novel coding sequences [26], chromatin organization [21], [27], and tissue-specific regulation [28] throughout mammalian evolution.

Previous studies have taken advantage of the high copy number of RTs to study their relationship with the host genome. These studies have begun to reveal the capacity of various genomic contexts to withstand retrotransposition. Within genes, certain regions are more susceptible to disruption by RTs than others [9]. These regions have been termed “hazardous zones,” and are highly correlated with exon density. Orientation of the RT with respect to the gene has also been determined to be an important factor in RT frequency. Endogenous retroviruses (ERVs) oriented in the sense orientation (SO) with respect to a gene are reduced within genes compared to ERVs in the antisense orientation (ASO) [29]. This pattern has also been observed in Alu and L1 [30], [31]. One hypothesis proposed for this antisense bias is that SO RTs contain sequence motifs such as those that alter splicing and polyadenylation [19], [29], and therefore are more deleterious in the SO [32]. In order to characterize the relationship between genes and inserted elements, we have examined the distribution of retrotransposons and their internal motifs within tissue-specific and housekeeping genes. We further explored the functional relationship with gene expression by examining whether polymorphic retrotransposons can act as expressed quantitative trait loci (eQTL) for co-localized genes.

## Results

### Recently inserted L1 and Alu exhibit an antisense bias that is uniform across gene deciles

**Figure 1:**
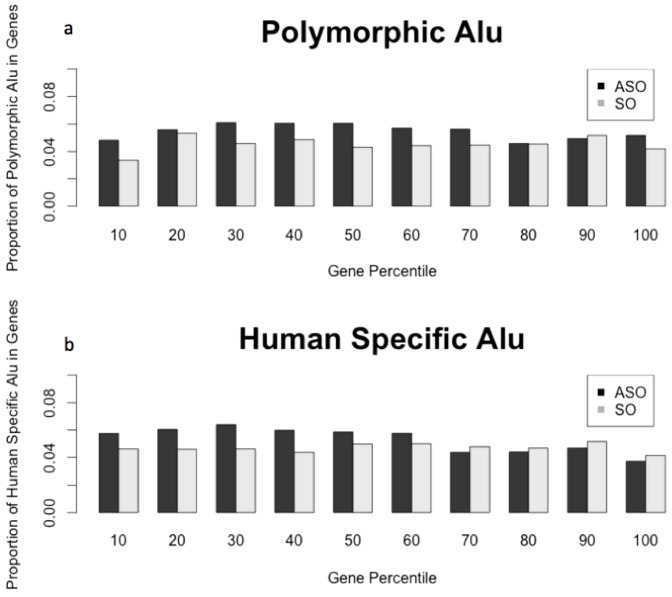
Young ASO and SO Alu show an initial antisense bias in genes. Polymorphic Alu [35] (a) and Human-specific Alu [36] (b) were subdivided into elements which were antisense oriented (ASO, black bars) or sense oriented (SO, grey bars) with respect to the colocalized gene. Counts of these Alu were tabulated within each gene decile for all genes and normalized by the total number of polymorphic or human-specific Alu in all genes. Student's t-test for polymorphic ASO vs. SO Alu p<0.001, human-specific ASO vs. SO Alu p<0.06.

The distribution of retrotransposons throughout the human genome shifted towards a non-uniform pattern over evolutionary time [1], [33], [34]. As mentioned previously, L1 elements are enriched within GC-poor regions, while Alu are commonly found in GC-rich regions [12], [13]. A second pattern of distribution emerges when analysis is restricted to gene regions. For example, previous studies have identified an increase of many families of retrotransposons near 5′UTRs [31].To distinguish whether this non-uniform distribution of retrotransposons within genes is a result of a bias during insertion, as opposed to an accumulation over time, we calculated the distribution of relatively recent Alu insertions within genes. Two previously published datasets were used for analysis: a) a group of polymorphic Alu from the 1000 genomes project [35], and b) Alu which are specific to the human lineage [36] (Dataset S1). We used all RefSeq genes and normalized for the difference in length by dividing each gene into ten bins of equal size (deciles). The frequencies of group (a) and group (b) were then calculated within each bin. As expected under a model of uniform integration, the proportion of polymorphic RTs were not significantly different from a uniform distribution (chi-square p-value>0.19) (figure 1a), although, contrary to expectation, there was an increase in the antisense bias compared to all reference Alu elements (Avg.:1.21, Std. Dev.:0.16). Human-specific Alu, which include RTs fixed within the population and are on-average older than polymorphic RTs, began to exhibit accumulation in both the antisense orientation (Avg.: 1.22, Std. Dev.:0.19), as well as a bias in distribution towards the first half of the gene (figure 1b). These results are largely consistent with previous findings from cell culture studies suggesting that some degree of antisense bias is introduced at the actual point of physical integration into the genome, separate from and in addition to any subsequent action of natural selection.

### Genic antisense bias is established soon after insertion and remains relatively constant over longer evolutionary time periods

Given that polymorphic Alu and human-specific Alu already appear over-represented in the antisense orientation, we next wanted to determine how this antisense bias might be modified over time. To address this question, we examined the rate of change in both L1 and Alu frequencies within genes and the ratio of ASO/SO over time. All L1 and Alu annotated in the Hg19 reference genome were used for this analysis. As a proxy for time, RTs can be ranked by relative age of retrotransposition. To order subfamilies in approximate order of subfamily age, we made use of the average nucleotide distance of subfamily members to their consensus sequence (Dataset S1). As detailed further in methods, the absolute rank order is most reliable at longer time scales, while at shorter timescales parallel amplification and additional non-sequential processes can lead to ambiguity in rank order. A list of ranks used in this analysis is presented in dataset S2 (see methods for further detail). Note that rank was ordered from smallest to largest levels of divergence, corresponding to more recent to more distant time periods. Hence an increase in enrichment when viewed in ascending rank order corresponds to a decrease in genic enrichment from older to younger element insertions. Results for each element class examined are described below.

#### L1

**Figure 2:**
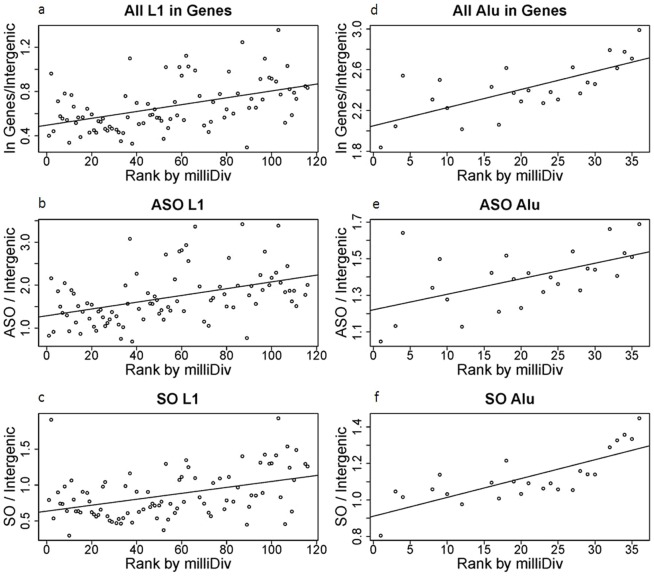
Alu and L1 densities increase in genes over time. Densities were calculated per subfamily as bp repeat element per bp region normalized by calculations for the same subfamily intergenically. Groups were divided into: All L1 within genes (a), antisense oriented L1 in genes (ASO L1) (b), sense oriented L1 in genes (SO L1) (c), all Alu within genes (d), antisense oriented Alu in genes (ASO Alu) (e), and sense oriented Alu in genes (SO Alu) (f). Subfamilies were then ranked based on their average base mismatches from consensus in parts per thousand (milliDiv) with an increase in milliDiv indicating a higher divergence, and therefore larger numbered rank (see Dataset S2). Results were plotted as an increase of this subfamily rank. The resulting slopes of density over subfamily rank were: 0.003_all L1_, 0.008_L1 ASO_, 0.004_SO L1_, 0.018_all Alu_, 0.008_Alu ASO_, and 0.010_Alu SO_.

**Figure 3:**
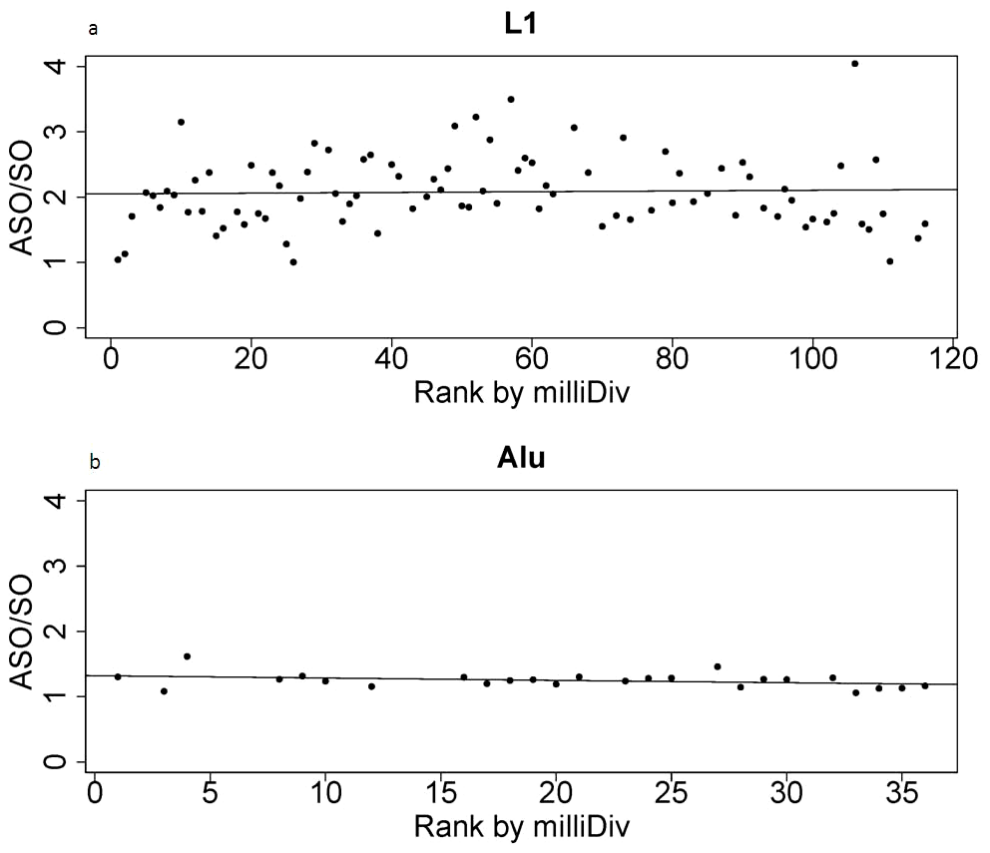
ASO/SO ratio remains consistent over time. Counts of all antisense oriented L1 in genes (ASO-L1) were compared to counts of all sense oriented L1 in genes (SO-L1) (a) and the counts of all antisense oriented Alu in genes (ASO-Alu) were compared to counts of all sense oriented Alu in genes (SO-Alu) (b). The resulting antisense to sense ratio (ASO/SO) was plotted by the subfamily rank as detailed in figure 2. Slopes of ASO/SO over subfamily rank were 0.0005 and −0.004 for L1 and Alu respectively.

We first separately examined the frequency of L1 elements in each orientation across time. Most L1 subfamilies were depleted within genes (figure 2a–c), with the exception of the oldest subfamilies, which were enriched compared to intergenic frequencies. When ordered by rank, the frequency of L1 subfamilies within genes increased (from the youngest to the oldest elements) along a linear trend (table 1). That is, the longer L1 families resided in the genome, the more enriched they were found to be among genes. The majority of L1 in the sense orientation were under-represented within genes, while the majority of subfamilies of antisense L1 were over-represented in genes. Both the sense and antisense slopes were positive over increasing rank, although with slightly different magnitudes (SO: 0.004; ASO: 0.008). We next examined the degree of genic antisense bias among subfamilies that amplified at different periods in time. Despite the difference in magnitudes of slope among SO and ASO elements indicated above, there remained relatively consistent levels of ASO/SO ratios across time (figure 3a). This relatively constant ratio over extended evolutionary time periods suggests that the establishment of antisense bias occurs proximal to, or at the point of, insertion and is essentially stable afterwards.

**Table 1:** L1 and Alu frequencies increase in genes over time.

	R^2^	F-test p-value	slope
**L1**	0.21	3.67E-06	0.003
**L1 ASO**	0.18	2.16E-05	0.008
**L1 SO**	0.17	4.01E-05	0.004
**Alu**	0.48	7.00E-05	0.018
**Alu ASO**	0.26	4.86E-03	0.008
**Alu SO**	0.58	4.74E-06	0.01

Counts of all L1 (L1), all Alu (Alu), antisense oriented L1 (L1 ASO), sense oriented L1 (L1 SO), antisense oriented Alu (Alu ASO), and sense oriented Alu (Alu SO), were tallied as bp element per bp region and normalized by intergenic calculations on the same subfamilies (plotted in figure 2). Linear regression was performed on these values versus the rank (see methods) and significance tested using an F-test.

#### Alu

In contrast to L1, the majority of Alu were over-represented in genes (figure 2d–f). After grouping based on subfamily, and consistent with previous studies, it was noted that the frequency of Alu increased linearly over increasing rank (table 1). Both ASO and SO Alu were over-represented within genes, except for young subfamilies in the SO. Similar to L1, the sense and antisense Alu slopes were positive over time but with similar slopes (SO: 0.010; ASO: 0.008) and a consistent ASO/SO ratio across time (Avg.: 1.24 ASO/SO, Std. dev.: 0.11) (figure 3b).

Combined with the L1 data, these results indicated that L1 and Alu density within genes had increased over the course of evolutionary time in relation to intergenic frequencies, but the overall level of antisense bias remained largely unchanged.

### L1 and Alu Antisense bias exhibits a linear decay across gene deciles and is inversely proportional to exon density

**Figure 4:**
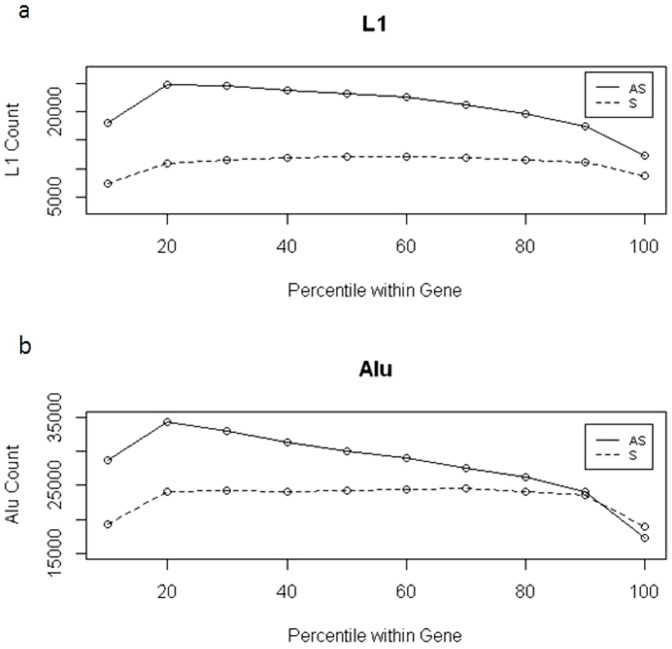
ASO bias decreases linearly across a gene. All L1 (a) and all Alu (b) annotated in UCSC Genome Browser RepeatMasker track (Hg19) were used in this analysis. Raw counts of L1 and Alu were tabulated for each gene decile over all RefSeq genes and subset based on orientation with respect to the colocalized gene. ASO = Antisense oriented (sold lines), SO = Sense Oriented (hashed lines). Slopes of the retrotransposon count over gene decile were calculated excluding the first and last bins, −99.4_L1 ASO_, 3.3_L1 SO_, −141.11_Alu ASO_, −2.7_Alu SO_.

To determine how RT frequency was dependent upon an element's position in a gene, we calculated the frequency of all L1 and Alu within each gene decile both in the ASO and SO (figure 4a & b). We were able to reproduce previous findings that demonstrated an accumulation of ASO near the 5′ region of the gene [31]. By normalizing for gene length we were further able to determine that the decay of ASO L1 and Alu is linear across genes. Both ASO L1 and Alu began at a relatively high frequency after the first gene decile and declined at a linear rate across the length of the gene (table 2). The lower limits in ASO frequency were observed within the deciles containing the 5′- and 3′-UTRs (see below). As indicated previously, we observed that human specific Alu (youngest class) were slightly biased for the ASO.

**Table 2:** ASO Alu and L1 decay across gene deciles while exon density linearly increases.

	N, in genes	slope, elements per decile	R^2^	F-test p-value
**L1, ASO**	208098	−99.36	0.91	2.00E-04
**Alu, ASO**	281711	−141.1	0.99	5.53E-08
**Exon, CDS**	497477	606.7	0.99	1.20E-07
**L1, SO**	109206	3.3	0.03	6.92E-01
**Alu, SO**	231331	−2.7	0.05	6.08E-01

Counts of antisense oriented L1 (L1, ASO), antisense oriented Alu (Alu, ASO), sense oriented L1 (L1, SO), sense oriented Alu (Alu, SO), and coding exons (Exon, CDS) were tabulated within all genes over the internal linear deciles (2^nd^–9^th^). Linear regression was performed on these counts to obtain the slope, R^2^, and F-test p-values.

Here we further note that the slope of human-specific ASO Alu was significantly different from the slope calculated with all Alu (Z-score: 2.07; p<0.03), indicating that the decay in antisense bias across genes most likely arose over evolutionary time. Alternatively, and somewhat less parsimoniously, recently inserted elements may have significantly altered their insertion pattern compared to older elements due to shifts in RT targeting mechanisms.

**Figure 5:**
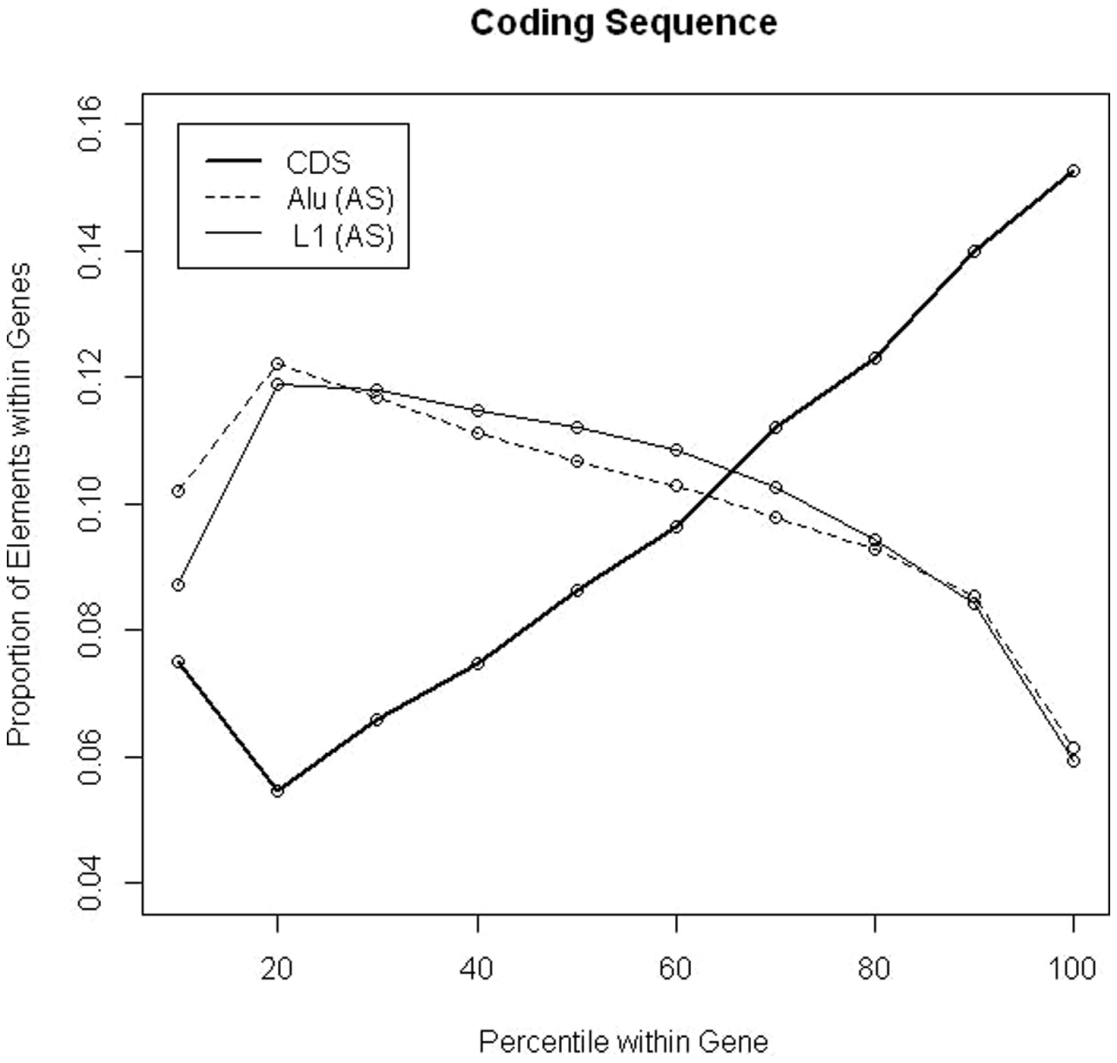
Exon density increases linearly across a gene. All CDS exons within the UCSC Genome Browser RefSeq track (Hg19) were used for this analysis. Counts of CDS exons were tabulated per decile in all RefSeq genes and normalized by the total number of CDS exons in all genes (solid bold line). The antisense oriented (AS) frequencies for L1 (solid thin line) and Alu (broken thin line) are included to exhibit the inverse relationship between antisense distribution and CDS density (Pearson's correlation coefficient: −0.94, p<5.21e-05).

Since previous studies have shown that ASO RT density was inversely correlated with exon density, we next calculated the frequency of coding exons (CDS) within each decile. Indeed, CDS density increased at a similar linear rate across gene deciles (figure 5 and table 2). The first decile of genes contained more CDS than the second decile. This was likely due to using the TSS as a starting point for calculation, which would enrich for 5′UTRs and consequently the coding exons that may be associated with them. Therefore, as has been indicated previously [9], ASO-RTs were inversely correlated with exon density (Pearson's Corr. value: −0.94; p-value: 5.21e-05) (table 3). Moreover, the distributions of exons and RTs were found to be linearly increasing and decreasing across a gene, respectively, indicating a predictable pattern of RT density within gene bounds.

**Table 3:** ASO Alu correlation with exon density is reduced in tissue-specific gene sets.

Gene Set	Genes (n)	Correlation with Exon Density	
		*ASO, Alu*	*SO, Alu*
**Housekeeping genes**	2237	−0.96	−0.40
**All genes**	42582	−0.94	−0.30
**Blood - neutrophil**	231	−0.76	−0.21
**Muscle**	111	−0.53	0.02
**Cartilage**	133	−0.53	0.23
**Testis**	252	−0.51	−0.38
**Liver**	305	−0.43	−0.22
**Colon**	122	−0.16	0.03
**Pancreas**	125	−0.08	−0.47

The distributions of Antisense Oriented Alu (ASO, Alu), Sense Oriented Alu (SO, Alu), and Coding Sequence (CDS) were calculated within each subset of genes. The Pearson correlation coefficients for Alu and CDS are presented for each gene set in the respective cells.

### RT frequency is reduced near 5′-UTRs

Although both are conserved, coding exons and UTRs have very different roles in the creation of a final gene product. It has been shown that exon density is highly predictive of RT frequency; however it is also possible that the 5′-UTR has a specific effect on RT abundance that is distinct from the effect imposed by coding exons. We therefore wanted to determine if the presence of a 5′-UTR was associated with altered RT frequency beyond what would be expected from exon enrichment alone.

**Figure 6:**
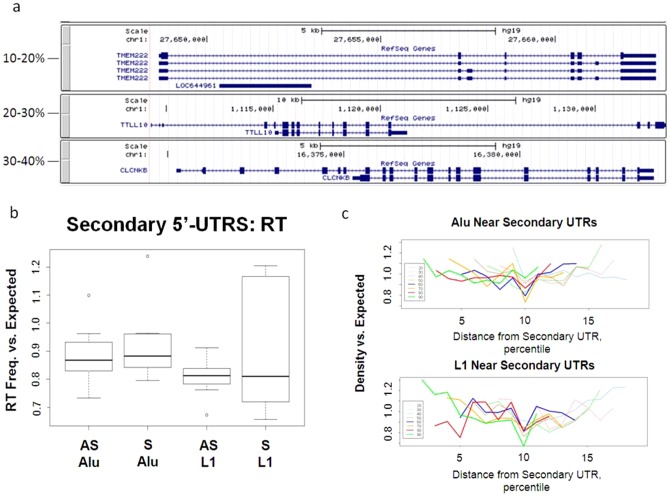
Frequency of L1 and Alu is reduced in the presence of a 5′-UTR. a) Examples of genes containing a secondary 5′-UTR within the gene boundaries. Each group shows an example of the secondary 5′-UTR occurring in a separate decile (examples shown: 1^st^ decile: TMEM222, chr1: 27648636–27662891, 2^nd^ decile: TTLL10, chr1: 1109286–1133313, and 3^rd^ decile: CLCNKB, chr1: 16370231–16383821). Screenshots were taken from UCSC Genome Browser (Hg19). b) Frequencies of Alu and L1 were then calculated within each decile of the aforementioned genes. These values were compared to expectation based on the calculations in the respective decile across all genes. Student's t-test p<2.99 E-06_L1 AS_, 0.04_L1 S_, 0.48_Alu AS_, 0.24_Alu S_. c) Antisense oriented Alu and L1 frequencies were calculated in all deciles for each group of genes that were separated as in figure 6a. These frequencies were compared to the expected frequencies based on overall values. Each line represents the change from expected for each gene group. The first and last deciles were excluded from analysis. 2^nd^ decile (light blue line), 3^rd^ decile (pink line), 4^th^ decile (light green line), 5^th^ decile (tan line), 6^th^ decile (blue line), 7^th^ decile (orange line), 8^th^ decile (red line), 9^th^ decile (green line).

In our method of analysis the first and last deciles consistently contained a 5′- or 3′-UTR, whereas the internal deciles were variable as to the presence of a coding exon. The resulting frequency plots revealed that RTs were highly reduced within the first bin (containing the 5′-UTR) in comparison to the second bin. Since this could be a result of a decrease in the density of CDS exons as well as an altered effect by the 5′-UTR, we needed to distinguish the effects from one another. To accomplish this, we restricted analysis to genes that contained a second 5′-UTR in each decile. Examples of genes that contained a secondary 5′-UTR are depicted in figure 6a.

Genes containing secondary 5′-UTRs were further grouped into subsets based on the decile location of the second UTR. For example, Group I genes contained all secondary 5′-UTRs in the first decile, calculated as the first 0%–10% of the gene. Group II contained genes where the secondary 5′-UTR was in the second decile (10%–20% of the gene), and so on for all ten deciles (see methods). The frequencies of all SO and ASO L1 and Alu were then calculated within these gene groups and compared to expectation based on whole genome averages. Both Alu and L1 were reduced in frequency versus expected though only L1 were significantly reduced (Student's t-test p: 2.99 E-06_L1 ASO_, 0.04_L1 SO_, 0.49_Alu ASO_, 0.24_Alu SO_) (figure 6b).

Across all genes, we have shown that the second decile, that neighbours the 5′-UTR containing decile, contains the largest number of L1 and Alu. We therefore tested if L1 and Alu were similarly increased in the deciles surrounding the secondary 5′-UTRs. We found that the L1 and Alu were also reduced to a lower extent in the bins surrounding the 5′UTR (figure 6c). L1 near secondary 5′-UTRs were depleted at a higher level than Alu, though this difference was not significant (Student's t-test p = 0.07). Most importantly, the change in exon density due to the altered binning procedure was not correlated with the effect of depletion on RTs (Pearson's correlation coefficient: −0.42 L1, −0.48 Alu; p = 0.30 L1, 0.34 Alu). This indicated that the reduced frequency of RTs near 5′-UTRs was not attributable to a correlation with the hazardous zone near an exon, but to the 5′-UTR itself.

### Antisense bias and correlation with exon density is reduced within tissue-specific genes

When taken as a group, RT antisense bias decreased linearly across gene bounds. Different functional groups of genes, however, contain varied numbers of RTs. For example, housekeeping genes have a predictably higher level of Alu frequency than other gene categories [37]. We therefore tested whether the antisense bias remained linear when subdivided by gene category. The effect of tissue-specificity was tested by correlating the distribution of Alu with the distribution of CDS within tissue-specific gene sets. The linear ASO slope remained present in housekeeping genes, but it was not maintained in tissue-specific gene sets. Furthermore, the correlation between ASO-RT frequency and CDS distribution was reduced within tissue-specific gene sets (table 3).

**Figure 7:**
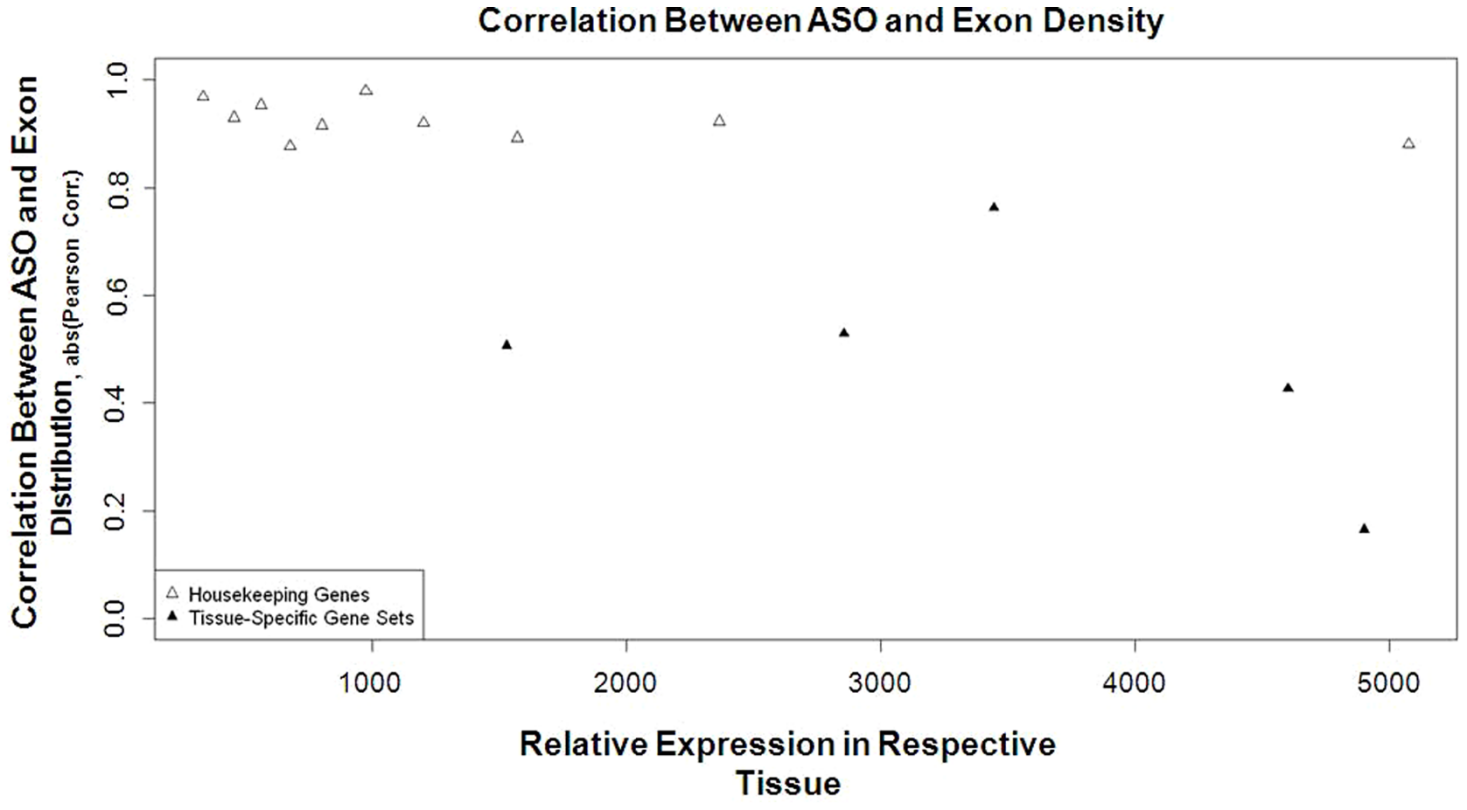
ASO bias is effected by tissue-specificity of the gene and not by relative gene expression. Genes were grouped by relative gene expression and tissue-specificity based on calculations from [57]. The Pearson's correlation coefficient was calculated between exon density and antisense retrotransposon frequency in each decile for housekeeping (empty triangle) or tissue-specific genes (filled triangle). Tissue-specific gene sets had smaller correlation coefficients than housekeeping genes and this effect was not modified by relative expression level in the respective tissue.

To determine if differences in gene expression across tissue types was confounding this result, we tested the correlation between ASO frequency and exon density as a function of relative expression in housekeeping and tissue specific genes. There was no link between expression level and the correlation between ASO and exon density (figure 7). Furthermore, since it has been shown that retrotransposons are restricted near constitutively expressed exons [38], we also wanted to check if the loss of antisense bias was affected by alternative splicing. Using the knownAlt annotation track from Hg19 (UCSC Genome Browser), we separated genes into those with known alternative splicing events, and those that are constitutively expressed. We found no significant differences between the distribution of ASO and SO between the two groups (chi-square p>0.25, p>0.23 respectively). We further calculated the correlation of ASO Alu density and CDS density within alternatively spliced genes. The negative correlation that is seen across all genes was replicated when examining only genes that are alternatively spliced (Pearson's correlation coefficient: −0.78, p<0.008).

### Frequency of functional binding motifs contained within Alu and L1 are dependent on gene category

Although the consensus sequences for Alu and L1 contain many functional DNA binding motifs, each individual RT in the genome is variable as to whether or not it contains these motifs. The loss or gain of a motif may occur by mutation at the nucleotide level or through retrotransposition of a parent element that has already lost or gained the motif sequence. To help determine how the functional motifs within Alu and L1 affect their frequency within genes, we analysed a subset of functional motifs found in the respective consensus sequences (table 4). The binding motifs presented in table 4 represent binding motifs for a variety of genes which have the potential to impact both retrotransposon as well as gene expression. For example, the 5′ portion of the polymerase III promoter (Pol-III) contains the A-box motif which is necessary for transcription factor binding and expression of Alu retrotransposons [39]. The YY-1 binding motif contained within L1 is both a component of the core promoter necessary for L1 transcription [40] as well as a general transcriptional repressor [41]. CpGs which are common in Alu and L1 can potentially effect levels of gene transcription [42] when methylated. Additionally due to the process of deamination, 5-methylcytosines are more likely to be mutated to thymines than non-methylated cytosines [43], and are commonly mutated in Alu [44]. The SRY motif is a binding domain for the testis-determining factor gene SRY which increases L1 transcription in 293 cells [45], and RUNX3 binding can act as both an activator and repressor of transcription and whose repression is linked with tumour progression [46]. Frequencies of each motif were calculated within genes and compared to intergenic proportions. When measured across all genes, the motifs present in table 4 were significantly depleted within genes when compared to intergenic frequencies. To test if the depletion of motifs was due to a general degradation of the whole element as opposed to the motifs themselves we measured the relative frequencies of the end region of Alu (Alu-terminus) that had no known binding partner when queried in JASPAR. This region showed no enrichment in either intergenic or gene regions.

**Table 4:** DNA binding motifs within L1 and Alu are depleted within genes.

		Depletion	
Motif	Sequence	Prop. in genes vs. Prop. Intergenic	Yates Corrected p<
Alu-Terminus	*gagactccgtctc*	1.00	NS
Pol III Promoter, Alu	*GGCCGGGCGCGGTGGCTCACGCC*	0.86	0.011
YY-1, L1	*AAGATGGCC*	0.76	0.015
CG, L1	*CG*	0.89	0.018
SRY, L1	*AGG/AT/CAAACAAAG/AC*	0.67	0.018
Polyadenylation, Alu	*AAATAAA*	0.95	0.026
RUNX3, L1	*TGAGG/AT*	0.81	0.062*
Polyadenylation, L1	*AAATAAA*	0.75	0.095*

Raw counts of motifs within Alu or L1 in genes or intergenically were normalized by the number of Alu or L1 within genes or intergenic regions. The presence/absence of the Alu-terminus motif was calculated as a negative control as it has not been shown to have a biologically significant impact on Alu expression or gene function (*/NS = Not Significant). The motifs presented in this table were depleted within genes compared to intergenic with a Yates corrected chi-square p-value p<0.05.

**Figure 8:**
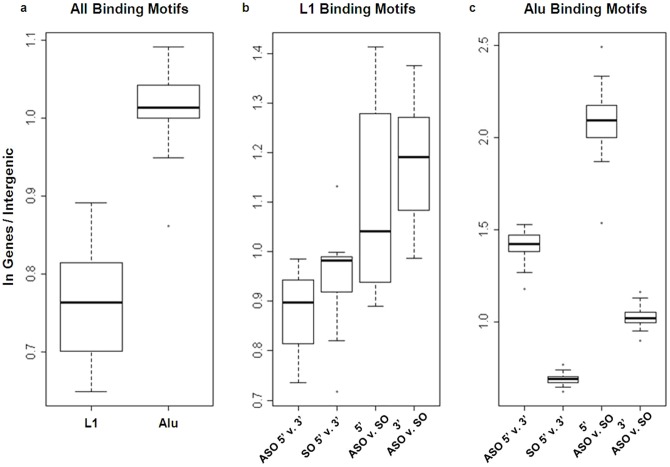
Depletion of motifs within RTs is dependent on position and orientation within the gene. The frequency in genes versus intergenic for all binding motifs (a), frequency of binding motifs contained within L1 (b), frequency of binding motifs contained within Alu (c). Calculations for b and c were restricted to the inner deciles with the largest and smallest antisense ratio respectfully, the 2^nd^ (5′) and 9^th^ (3′) deciles. The first two boxes show motifs present in the 2^nd^ decile compared to the same motifs in the 9^th^ decile for both the antisense orientation (ASO) and sense orientation (SO). The last two boxes restricted analysis to within the same bin and compared the motifs in the antisense orientation to the same motifs in the sense orientation.

Since we have shown that, at the level of entire elements, L1 and Alu frequencies are correlated with relative location within a gene, we examined the dependency of individual motifs on the relative location within the gene. To reduce the number of statistical tests (and hence required corrections), we restricted analysis to the bins that contained the extremes of Alu and L1 frequency. These were the first and last bins within the linear range of Alu/L1 frequency, which contained the most and least Alu and L1 respectively. Motif frequencies were normalized by the count of ASO or SO elements within the bin and then compared between bins (figure 8). Contrary to the general trend of L1 elements as whole, functional motifs within L1 were over-represented near the 3′ end of genes compared to the 5′ end. Similarly motifs within sense oriented Alu were also over-represented near the 3′ end of genes. Conversely, motifs within antisense oriented Alu were over-represented near the 5′ gene end. Overall, antisense oriented L1 contained more functional motifs than sense oriented.

#### Alu

To account for differences between gene groups we further classified subsets of genes into functional groups (housekeeping, tissue-specific) (table 5). The relative presence of CpGs did not change between housekeeping or tissue specific genes (L1 data not shown). However, when elements were present within housekeeping genes, Pol-III promoters were more often in the ASO and, conversely, they were more often in the SO for tissue-specific genes. The binding motifs for Brn2 and HNF4 were differentially represented depending both on the orientation within the gene and whether the sequence was located near the 5′ or 3′ end of the gene.

**Table 5:** Alu-contained DNA binding motifs within housekeeping and tissue-specific genes.

Housekeeping/Tissue Specific	Chi Square p<	In Genes/Intergenic	ASO/SO	ASO 5′/ASO 3′	SO 5′/SO 3′	5′ ASO/5′ SO	3′ ASO/3′ SO
**Total Elements**	NS	1.00	1.00	1.00	1.00	1.00	1.00
**CG**	NS	1.00	1.00	0.98	0.99	0.98	0.99
**Polyadenylation**	6.5E-03	0.92	0.98	1.08	0.99	0.97	0.89
**Pol III Promoter**	5.4E-18	0.88	1.67	0.86	0.51	1.56	0.92
**BRN2.03**	4.9E-07	0.94	0.93	0.97	1.45	0.76	1.14
**HNF4.02**	9.8E-53	1.22	0.77	0.57	0.78	0.63	0.87

The presence of Alu-contained DNA motifs was compared between housekeeping genes and tissue specific genes. The motifs presented here contained significantly different counts between housekeeping genes and tissue-specific genes using a chi-square test. Proportions were then compared within each category denoted in the table header. Motifs counts were tabulated for Antisense (ASO), Sense (SO), the first linear, 2^nd^, decile (5′), and the last linear, 9^th^, decile (3′). Since methylated cytosines have a higher rate of mutation than non-methylated CpGs and methylation may be different within genes and intergenically, CpG proportions were also included in the table even though they did not pass significance (*NS).

#### L1

Overall, the frequencies of motifs found within L1 followed slightly different patterns. Motifs within L1 were significantly depleted within genes compared to motifs within Alu (t-test: p<1.3e-05) (figure 8a). They did, however, follow the same global trend of frequency being dependent on orientation and location (figure 8).

These results indicate that not only are whole RT elements non-randomly distributed within genes, but also functional motifs within Alu and L1 are further depleted. Furthermore, this depletion is sensitive to orientation and location of the retrotransposon within the gene as well as the tissue specificity of the gene itself.

### Truncated L1 escape depletion near the 5′-UTR

**Figure 9:**
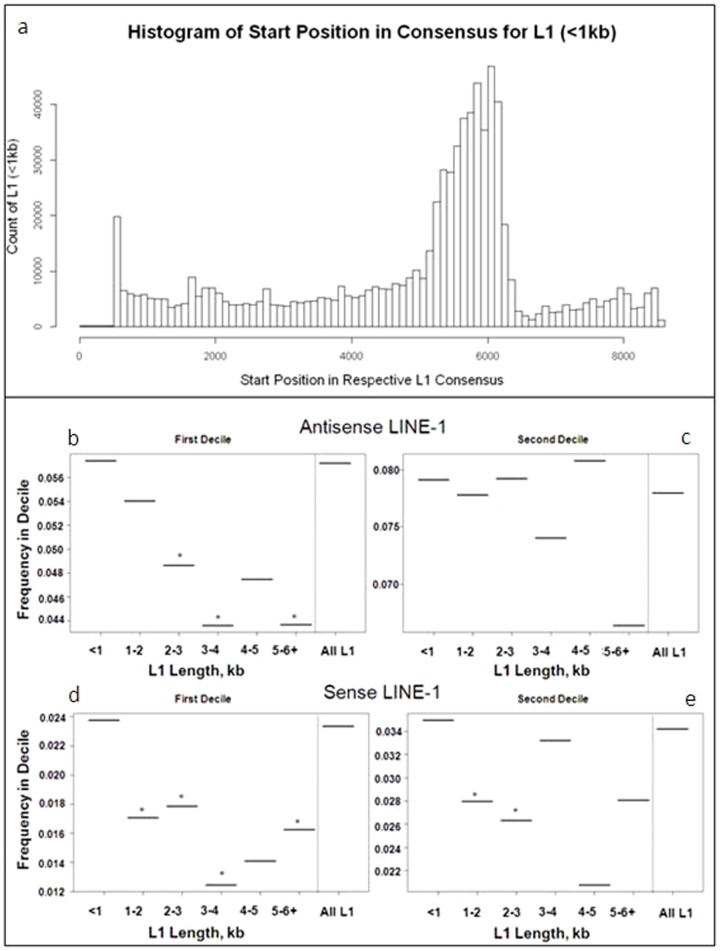
Full-length L1 are reduced near the 5′-UTR of genes. a) Histogram of the start position within the L1 consensus sequence of L1 that are less than 1 kb. The L1 promoter lies between approximately 1–1000 bp depending on the consensus. The peak of where L1<1 kb start in the respective consensus sequence is shifted towards 6 kb. b–e) L1 were subdivided by length into groups of <1 kb, 1–2 kb, 2–3 kb, 3–4 kb, 5 kb>, or all L1 within genes. Frequencies of antisense (top row) and sense (bottom row) that fell within the first (left column) or second (right column) gene deciles were then calculated for each group of L1. L1 located in the first decile of genes showed a reduced frequency with increasing size of the element for both antisense and sense L1 (* = Fischer's two-sided p-values significant after Bonferroni correction).

The human L1 consensus sequence is approximately 6 kb, although it varies by subfamily. During retrotransposition L1 are often truncated, resulting in an average size of 900 bp [1]. Reverse transcription of the RNA template initiates near the 3′ end of RNA and often the L1 retrotransposase is unable to complete retrotransposition of the entire element into the insertion site, resulting in truncation of the 5′-end [47], [48]. In the human genome L1 are variable in size, a majority of which are less than 1 kb (table 6). The 5′ region of L1, which is often lost during truncation, contains the Polymerase II promoter which can effect expression of neighbouring genes [19], [20]. To identify whether L1 (<1 kb) lack the promoter region, we determined the start position of each L1 (<1 kb) within the respective consensus using local RepeatMasker (figure 9a). The results indicate that a majority of these small L1 begin after the canonical promoter sequence.

**Table 6:** Size range of L1 in the human reference genome.

Size of L1 Element bin, kb	Count
1	833043
2	76631
3	20349
4	8174
5	3787
6+	10181

The counts of L1 within each size range (<1 kb, 2 kb, 3 kb, 4 kb, 5 kb, 6 kb>) were tabulated using annotation from UCSC RepeatMasker track (Hg19).

To test whether the linear decay of antisense oriented L1 was related to the size of the element, we calculated the proportions of L1<1 kb, 1–2 kb, 3–4 kb, and 5 kb+ versus expectation based on all L1. Fischer's exact test p-values were calculated between the count of observed size-specific L1 and the expectation within bins 1 and 2 since these bins had the largest effects of antisense bias. We found that full-length L1 (5 kb+) were greatly reduced in frequency near the 5′ region of the gene compared to expectation (figure 9). This was true of L1 in both orientations. Conversely small L1, <1 kb, were increased near the 5′-UTR. The small sample size for the full length L1 in the second decile may be playing a role in their failure to pass significance after Bonferroni correction.

### Polymorphic Retrotransposons can act as eQTLs

Putku et al. have previously linked the presence of polymorphic RTs to differential gene expression of the WNK1 gene [49]. To demonstrate this concept on a genome-wide level, we correlated the presence or absence of polymorphic RTs with expression in the same genes. Polymorphic retrotransposon locations were taken from the 1000 genomes dataset [35] and correlated with gene expression from GEO dataset E-MTAB-197 [50] (see methods). We restricted analysis to loci where the inserted allele was present in at least 60 individuals. 616 polymorphic RTs were then tested for correlation with expression of the coincident gene using Spearman's rho. This proof of principle analysis revealed 3 elements that were significantly correlated with differential gene expression after correction by FDR (table 7). The orientations and decile locations of these elements within the respective gene are tabulated in table 8. At all 3 loci, the presence of the RT insertion had negative effects when correlated with gene expression. The significantly correlated polymorphisms were all Alu elements, which are the smallest and most prevalent of the queried elements. Although this is too small of a sample size to infer the relationship between orientation and effect on gene expression, it is a proof of principle that a subset of polymorphic RTs which are present in the population are correlated with gene expression levels.

**Table 7:** Effect of retrotransposon eQTLs on gene expression.

Gene*	Retrotransposon*	Variant*	Effect	rho	p	FDR corrected q-value	AF* (CEU|YRI|CHBJPT)
**IPP**	AluYk1	Absence	−0.50	−0.48	1.23E-04	0.041	0.61|0.77|0.62
**GNS**	AluYa5	Absence	−0.38	−0.48	1.55E-04	0.041	0.39|0.05|0.16
**RAB11FIP3**	AluSp	Absence	−0.43	−0.46	2.45E-04	0.043	0.54|0.90|0.77
**SRP68**	Alu	Presence	−0.31	−0.44	4.71E-04	0.055	0.19|0.41|0.39
**TULP4**	AluYb8	Absence	−0.43	−0.44	5.34E-04	0.056	0.39|0.31|0.62

Retrotransposon polymorphisms were correlated with gene expression from microarray dataset E-MTAB-197. The top significant effects after FDR correction are presented. Gene: Gene within which the polymorphism occurred and whose expression is correlated with the presence/absence of the polymorphism; Retrotransposon: Polymorphic retrotransposon identified by the 1000 genomes project; Variant: Noted as the presence or absence versus the reference genome; Effect: The effect was calculated by regressing variant status on normalized microarray expression level; rho: Spearman's rank correlation rho was calculated for each comparison; p : raw p-value from Spearman's rank correlation; AF: Allele frequency in given populations;

* denotes values which are taken directly from 1000 genomes data [35].

**Table 8:** Location of retrotransposon eQTL within gene.

Gene*	Retrotransposon*	Orientation	Decile	Proximity to Closest Exon, bp
**IPP**	AluYk1	Sense	2	282
**GNS**	AluYa5	Antisense	4	358
**RAB11FIP3**	AluSp	Antisense	1	1749
**SRP68**	Alu	Sense	2	201
**TULP4**	AluYb8	Antisense	7	2774

Polymorphic retrotransposons that were significantly correlated with differential gene expression (see table 7) are presented here. Retrotransposon: Family or subfamily, where known, of the polymorphic transposon. Orientation: Direction of retrotransposon transcription with respect to gene transcription; Decile: The decile within the gene that the polymorphism occurs; where decile 1 contains the transcription start site and decile 10 contains the transcription stop site.

* denotes values which are taken directly from 1000 genomes data [35].

## Discussion

These results highlight the presence of predictive relationships between retrotransposons and the genes in which they reside. Primarily, we identified a linear decay of ASO RTs across the length of a gene that was correlated with exon density. The failure to observe a similar decay in polymorphic elements suggests this distribution is not present at the initial time of insertion and instead developed as a result of selective pressure relatively soon after insertion. The extent of antisense bias (ASO/SO), independent of gene decile, remained constant over time although the overall frequencies of RTs in genes steadily increased with increasing age. This is supported by previous cell line work which has shown that Alu may exhibit preferential insertion in the antisense orientation within a gene [51].

One factor strongly affecting the linear decay of ASO L1 retrotransposons is the length of the element. L1 that were truncated to <1 kb were substantially increased in frequency near the 5′-UTR when compared to full length L1. Although we postulate here that this was likely due to the size of the L1, it is also possible that the presence or absence of the bidirectional Pol-II promoter plays a role in maintenance of L1 near the 5′-UTR. Our analyses here cannot readily disentangle the effects of length, promoter presence/absence, and presence of motifs along the length of the element. Additional subfamily-specific analyses, adjusting for variability in promoter positions among L1 consensus sequences, will be required to tease apart these variables. Ideally, such analyses would be accompanied by experimental data examining the impact of various L1 insertion scenarios.

Contrary to previous findings, RTs near 5′-UTRs were reduced in frequency, most significantly for L1 in the antisense orientation. Our analysis was capable of distinguishing this trend by removing the confounding effect of low exon density that is commonly present near 5′UTRs. Since the linearly decreasing antisense bias is highly correlated with CDS exons, this indicates that the commonly reported increase in antisense oriented RTs near the 5′-UTR is more likely due to a lack of CDS exons as opposed to a function of the UTR itself, while the overall reduction in elements in either orientation is attributable to the UTR itself.

As previously reported, we observed a relative increase of RTs within genes over time. We consider it most likely that the increase in relative frequency of subfamilies within genes compared to intergenic is not a result of population fixation within genes, but, rather, a decrease of element numbers within intergenic regions. This could be explained by the relaxed purifying selection against deletion events within intergenic regions compared to genic [52]. Such deletions would remove elements from these intergenic regions at a higher rate than within genes. However, we cannot exclude positive selection as an explanation for genic enrichment, nor do the analyses described here provide direct evidence for the postulated biased loss of intergenic elements. Additional work is necessary before more definitive statements regarding the role of positive, as opposed to purifying, selection in shaping RT distributions within genes.

One of the primary results of this study indicated that the bias towards antisense RTs near the 5′ UTR and the decay of bias toward the 3′ region of the gene occurred relatively soon after insertion, while the elements remained polymorphic within the human population. This was evidenced by the observation that the linear decay from 5′ to 3′ of the gene was not evident from more recently inserted, polymorphic elements. As noted above, we did not, however, detect a difference in the overall genic ASO/SO ratio between the polymorphic and non-polymorphic elements. This discrepancy could be attributed to the fact that the change in slope of decay was a more sensitive measurement than overall bias across the gene. If this explanation is correct, additional polymorphic element data from population screening should ultimately allow for the overall ASO/SO genic bias to be detectable. Based on our results the selective bias towards a linear decay of antisense orientation appears to be separate from the antisense bias introduced during the insertion process itself.

One possible avenue, through which polymorphic elements may be influencing fitness, is through acting as eQTLs. It remains to be determined, however, if the elements in this case are directly impacting expression levels or simply tagging another functional variant in linkage disequilibrium with the element. The trends in RT distribution observed here may be helpful in predicting the effects of RTs insertions on a co-localized gene. Factors identified to be important in RT distribution were RT type, orientation with respect to the gene, proximity to a CDS, proximity to a 5′-UTR, and tissue specificity. While the biological mechanisms underlying these observations remain undetermined, it is plausible that the juxtaposition of ASO elements near intron-exon junctions interferes with splicing regulation [38], [53], [54], as previously proposed. Here, we further show that the extent of this effect is dependent on tissue specificity of gene expression.

## Methods

### Data Sources

Retrotransposon (RepeatMasker) coordinates were downloaded from the UCSC genome browser (Human, hg19). Polymorphic Alu were taken from the supplemental data in the original publication [35]. Polymorphic mobile elements labelled as deletions, or those missing strand information, were removed from the dataset. The resulting elements were converted from Hg18 to Hg19 coordinates using the Lift Over tool from UCSC Genome Browser. Those elements that could not be converted were excluded from analysis. Human Specific Alu were taken from the supplemental data in Bennett et al. Alu were converted from Hg18 to Hg19 coordinates using the Lift Over tool from UCSC Genome Browser. Those elements that could not be converted were excluded from analysis.

### Binning Reference Locations

Gene (RefSeq flat file track), and Retrotransposon (RepeatMasker) coordinates were downloaded from the UCSC genome browser (Human, hg19). Each gene was subdivided into ten bins of equal size where bin1 began at the transcription start and decile 10 ended at the transcription stop. This method of binning resulted in a concentration of exons in the first and last bins. Further calculations took this bias into account. Repeats analyses were limited to recent inserts only where mentioned.

### Determining Ranks for Retrotransposon Subfamilies

L1 were ranked first by subfamily group by the average divergence from their respective consensus sequences, as calculated in [55]. Alu were also ranked by average nucleotide distance of each family from its respective consensus. In the case of Alu elements, the Poly-A tail was first removed, and only those elements with equivalent base pair length to their consensus sequences were included in the analysis. The average number of nucleotide differences among all consensus-element comparisons was then tabulated as used for rank ordering. The applied rankings very closely followed the Alu subfamily naming convention. This approach follows conventional methods for dating retrotransposon families in many respects. It does, however, have its limitations, particularly over shorter timespans (i.e., levels of nucleotide divergence). We consider this approach more conservative as the timescale grows larger because we make no assumptions about mutation rates over time or across element sequences. The only assumption made is that mutations are gradually accumulated over time, and this should hold true in the majority of instances, barring gene conversion and rare substitutions that revert nucleotides back to the corresponding consensus base. As the purpose of our ranking is to show no systematic increase or decrease in ASO/SO ratio over longer evolutionary periods, this relative ranking method should be suitable for our purposes. Over shorter timescales, however, it fails to capture the variance in element age estimates, as well as the fact that subfamilies can propagate over extended periods of time and can often do so in parallel with other subfamilies. In this sense, considering rank positions as distinct time points is a gross oversimplification. As our purpose in this particular analysis is to examine large scale trends in the magnitude of antisense bias, however, we do not expect these simplifications to impact our results or interpretation.

### Identifying Genes with Secondary UTRs

5′-UTR coordinates were selectively downloaded from the UCSC genome browser RefSeq Gene track. These coordinates were intersected with gene deciles to determine which 5′-UTRs were within gene boundaries. Genes were then grouped depending on which decile the 5′-UTR was located within.

### Distribution Analysis

Counts of elements within each decile were tabulated using an in-house script that intersected gene locations with RepeatMasker annotations to tabulate strand orientation counts per decile. Frequencies of elements within deciles were defined as the count of elements in a decile (for all genes) per total elements (across all genes). For SO-only or ASO-only plots, subfamily counts were normalized by the intergenic frequencies of the respective subfamilies in order to account for variable amplification rates. Trend lines were obtained via linear regression. For the analysis of L1 and Alu maintenance in genes as a function of rank by milliDiv; milliDiv was calculated as the average base mismatches in parts per thousand, as tabulated in RepeatMasker (UCSC Genome Browser, Hg19). A minimum sample size for comparison between subfamilies was calculated using G*Power3 [56], using the Chi-Square test: Goodness-of-fit tests: Contingency tables test with an effect size of 0.1. This resulted in a minimum sample size of 1545, which we used as a minimum element count threshold for inclusion in subsequent linear regression analysis. To calculate the slopes of all Alu, L1, and exon counts across gene deciles, linear regression was performed on the internal deciles (i.e.: 2–9). Leading and trailing deciles were excluded to reduce the introduction of bias attributed to an “exon anchoring effect.” Briefly, by using the transcription start and stop positions as gene boundaries, the first and last deciles will always contain a minimum of one exon each due to standard gene biology. Therefore, counts in these deciles are systematically skewed and less representative of the general pattern across the gene.

### Comparison of Frequency in Genes versus Intergenically

To correct for differences in genomic coverage within genes and intergenically, retrotransposon frequencies were calculated as a factor of their total length as opposed to count. Coverage in bp was tabulated for each element and summed per subfamily. Alu subfamilies were ranked according to age as per presence in humans.

The values within genes were then divided by the total bp covered by similar elements intergenically. All values were normalized by the total bp in the queried region.

### Tissue Specific Gene Sets

Gene categories were grouped based on the calculations in [57]. Power calculations resulted in the restriction of analysis to tissues with >100 genes. Decile and frequencies were performed as above, but with the restricted gene sets.

### Motif Analysis

To reduce multiple testing we restricted motif analysis to sequences that have been previously identified in Alu or L1. Exact matches, unless otherwise stated, were searched in the necessary region (e.g.: in genes, intergenic) using an in-house program. Counts of each motif were tabulated in each region and, where necessary, were normalized by the relative element density. Truncated L1 were identified by comparing every known L1 (RepeatMasker, Hg19) versus known L1 consensus sequences using a local BLAST alignment. The start position of each L1 was denoted as the start position within its respective consensus sequence.

### Retrotransposon eQTL Analysis

Polymorphic retrotransposons were filtered from the dataset in [35] where polymorphisms were included if they resided within a gene, and if at least 60 individuals were positive for the polymorphism. Expression data was taken from the normalized microarray dataset [50]. Individuals that overlapped between both datasets were used for analysis. Expression values were correlated with variant status using Spearman's rank correlation rho followed by false discovery rate correction using the fdrtools package in R.

## Supporting Information

Dataset S1 **List of Polymorphic Retrotransposons and Human-Specific Retrotransposons adapted from** [**35**] **and** [**36**] **.**
(XLSX)

Dataset S2 **Ranked list of L1 and Alu subfamilies used in this study.**
(XLSX)
