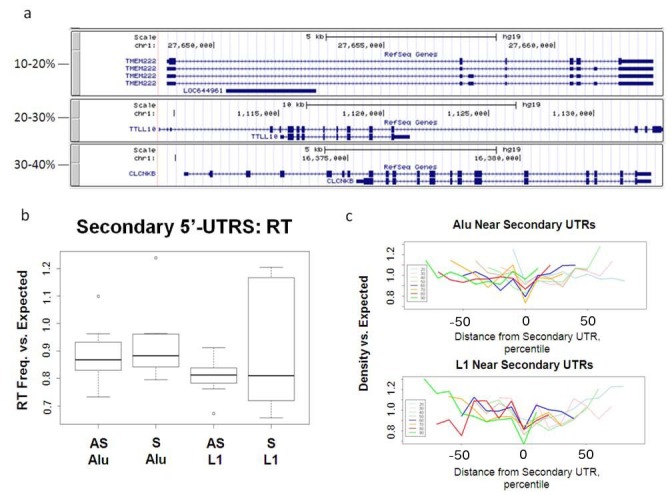# Correction: Linear Decay of Retrotransposon Antisense Bias across Genes Is Contingent upon Tissue Specificity

**DOI:** 10.1371/annotation/dfa05103-fc65-4f07-b30f-72a6e91613ff

**Published:** 2014-01-08

**Authors:** Sara Linker, Dale J. Hedges

Several errors are present in this manuscript.

The Corresponding Author's name is incomplete. It should be Dale J. Hedges.

The second P value listed in the Figure 1 legend is incorrectly listed as p<0.06. It should be p<0.03. The correct legend reads:

Polymorphic Alu [35] (a) and Human-specific Alu [36] (b) were subdivided into elements which were antisense oriented (ASO, black bars) or sense oriented (SO, grey bars) with respect to the colocalized gene. Counts of these Alu were tabulated within each gene decile for all genes and normalized by the total number of polymorphic or human-specific Alu in all genes. Student's t-test for polymorphic ASO vs. SO Alu p<0.001, human-specific ASO vs. SO Alu p<0.03.

The axis in Figure 6c is incorrect. Please see the corrected Figure 6 here: 

**Figure:**